# Mutual Information Neural-Estimation-Driven Constellation Shaping Design and Performance Analysis

**DOI:** 10.3390/e27040451

**Published:** 2025-04-21

**Authors:** Xiuli Ji, Qian Wang, Liping Qian, Pooi-Yuen Kam

**Affiliations:** 1 Institute of Cyberspace Security, Zhejiang University of Technology, Hangzhou 310023, China; 211122030028@zjut.edu.cn (X.J.); lpqian@zjut.edu.cn (L.Q.); 2 The School of Science and Engineering, The Chinese University of Hong Kong (Shenzhen), Shenzhen 518172, China; pykam@cuhk.edu.cn

**Keywords:** constellation shaping, high-order QAM, mutual information neural estimation, mutual information maximization

## Abstract

The choice of constellations largely affects the performance of both wireless and optical communications. To address increasing capacity requirements, constellation shaping, especially for high-order modulations, is imperative in high-speed coherent communication systems. This paper, thus, proposes novel mutual information neural estimation (MINE)-based geometric, probabilistic, and joint constellation shaping schemes, i.e., the MINE-GCS, MINE-PCS, and MINE-JCS, to maximize mutual information (MI) via emerging deep learning (DL) techniques. Innovatively, we first introduce the MINE module to effectively estimate and maximize MI through backpropagation, without clear knowledge of the channel state information. Then, we train encoder and probability generator networks with different signal-to-noise ratios to optimize the distribution locations and probabilities of the points, respectively. Note that MINE transforms the precise MI calculation problem into a parameter optimization problem. Our MINE-based schemes only optimize the transmitter end, and avoid the computational and structural complexity in traditional shaping. All the designs were verified through simulations as having superior performance for MI, among which the MINE-JCS undoubtedly performed the best for additive white Gaussian noise, compared to the unshaped QAMs and even the end-to-end training and other DL-based joint shaping schemes. For example, the low-order 8-ary MINE-GCS could achieve an MI gain of about 0.1 bits/symbol compared to the unshaped Star-8QAM. It is worth emphasizing that our proposed schemes achieve a balance between implementation complexity and MI performance, and they are expected to be applied in various practical scenarios with different noise and fading levels in the future.

## 1. Introduction

Recent times have witnessed a tremendous increase in network capacity requirements in the field of both radio frequency (RF) and laser-based satellite and optical wireless communications. And both capacity-approaching coding and power-efficient decoding techniques have become increasingly mature for combating noise impairments in RF or laser links [1,2]. In this context, capacity-approaching modulation formats, in addition to coding, have become the latest approach for improving communication capacity, due to their high spectral efficiency [3,4]. Note that ultra-bandwidth-efficient communications require higher-order modulation formats [5]. Even though higher-order quadrature amplitude modulation (QAM) is mostly used to increase data rates significantly, its sensitivity to link impairments such as additive white Gaussian noise (AWGN) also increases, which thus deteriorates transmission performance [6]. In this way, constellation shaping is imperative for coherent high-order modulated systems, including geometric constellation shaping (GCS) and probabilistic constellation shaping (PCS) [7,8,9,10,11]. To be specific, GCS involves the arrangement of point locations on a two-dimensional plane to optimize the constellation shape, in which traditional numerical optimization becomes more intricate as the order or dimension increases [8]. The alternative PCS adjusts the probability distribution with a fixed constellation shape to facilitate rate adaptation, which nevertheless necessitates an external distribution matcher for effective execution and leads to an increased complexity in the transceiver [10]. In addition, joint geometric and probabilistic constellation shaping (JCS) is also receiving attention for its better spectral efficiency [12,13].

It is worth noting that, in addition to the design metric of symbol error rate (SER) [12], mutual information (MI) is often used as a key metric to evaluate the maximum achievable information rate over different channels for most constellation optimization systems. And there is a substantial body of literature dedicated to MI maximization, to narrow the gap to the Shannon limit in pure AWGN [7,8,9]. Most works have considered the MI calculation using the underlying joint or conditional probability density function (pdf), given the specific channel model assumed. However, it is very challenging to directly and accurately evaluate MI, since the actual data are highly discrete and the specific distribution is unknown in practice [14]. However, most works have solely focused on traditional optimization methods, whose generalization is very limited due to strict formulations.

To address the above issues, effective deep learning (DL)-based signal processing strategies have been explored for many optimization tasks [15]. For example, refs. [16,17] used MI maximization as a visualization tool for maximizing parametric variational information and analyzing the *f*-divergence lower bound. More importantly, ref. [18] proposed mutual information neural estimation (MINE) as a deep neural network (DNN)-based parameter estimator, which employed gradient descent on the DNN to estimate the correlation between high-dimensional continuous random variables. Another prominent example is treating a communication system as an end-to-end (E2E) reconstruction optimization task, aiming to jointly optimize the transmitter and receiver components in a single process. E2E-based training systems have been adopted and proven to have excellent constellation shaping performance in limited works [19,20,21]. Specifically, ref. [19] designed an autoencoder for both constellation shaping and detection, employing strategic initialization to handle the highly non-convex constellation. Ref. [20] designed a GCS optimization scheme that exhibited robustness to uncertainties in channel conditions, including both AWGN and nonlinear interference noise. And [21] presented a novel E2E-based learning method of joint geometric and probabilistic constellation shaping for coded-modulation systems to maximize generalized MI.

This work thus proposes novel MINE-driven constellation shaping schemes, i.e., MINE-GCS, MINE-PCS, and MINE-JCS, to optimize high-order modulations using MI maximization for AWGN in terms of MI performance. Innovatively, the MINE network functions as a data-driven MI maximization module, by estimating the MI from abundant samples, without knowing the explicit channel information. And differently from the E2E structure, the MINE-driven schemes focus solely on optimizing the transmitter end and do not involve the decoder. Specifically, our contributions are summarized as follows:

- We propose novel MINE-driven high-order constellation shaping schemes, where a DNN-based MINE module is explicitly introduced to address the exact MI calculation problem. In MINE optimization, the MI is estimated by backpropagation from abundant samples without explicit knowledge of the channel information, which traditional methods struggle with.C Additionally, a DNN-based encoder and probability generator are employed to adaptively optimize the point locations and probability distributions with respect to the varying signal-to-noise ratio (SNR). Unlike the E2E system, our MINE-driven system does not involve the decoder, thus avoiding the training loss and computational complexity attributed to decoding.
- Our DNN-based deign is a lightweight model, due to the simple network structure involved on the transmitter side. Our MINE-driven schemes have less time and computational complexity than the E2E and other recent DL-based schemes. Note that our MINE-PCS has the least number of floating point operations (FLOPs) and parameters, while our MINE-GCS has the lowest training time per SNR.
- As expected, our MINE-driven schemes performed much better with AWGN over the studied parameter ranges of SNR of practical interest compared to the unshaped QAM and other DL-based schemes. For example, with a higher-order design, 64-ary MINE-GCS could achieve an MI gain of about 0.1 bits/symbol.

The remainder of this paper is organized as follows: Section 2 introduces the system model and the principle of MINE. Section 3 presents the specific MINE-driven constellation shaping schemes. Section 4 validates the MI performance via extensive simulations. Finally, Section 5 concludes this work.

## 2. System Model

This section introduces the signal model, the MI metric, and the MINE principle in detail, which are the basis for the constellation design.

### 2.1. Signal Model

A pure AWGN channel model is considered in our work, and thus the discrete-time, complex received symbol $y_{k}$ is given by

(1) $$y_{k}=x_{k}+n_{k},$$

where $x_{k}$ denotes the transmitted symbol chosen from *M*-ary modulated constellations in the *k*-th time slot, and $n_{k}$ denotes a zero-mean, complex Gaussian random variable with variance $N_{0}$. Here, the transmit SNR is defined as $\frac{E_{s}}{N_{0}}$, where $E_{s}$ is the average symbol energy. To account for shaping-induced rate differences, all mutual information values are reported in bits per channel use, ensuring a fair and consistent basis for comparison across different constellation structures.

### 2.2. MI Metric

To evaluate the performance of any memoryless channel, the maximum achievable information rate is considered here. Specifically, the MI denoted as I(X;Y) is formulated as [22]

(2) $$\begin{array}{cc} \;I(X;Y) & =H\left(X\right)-H\left(X|Y\right)\\ \; & ≜E\left[\log\frac{f_{Y|X}\left(y|x\right)}{f_{Y}\left(y\right)}\right], \end{array}$$

where H(X) represents the entropy and $H\left(X|Y\right)$ represents the conditional entropy. In addition, E[·] represents the mathematical expectation with respect to both *X* and *Y*, $f_{Y}$ represents the pdf of the received signal *Y*, and $f_{Y|X}$ represents the conditional pdf of *Y* given the transmitted signal *X*. Note that MI closely depends on the pdf $f_{Y|X}$, and the nature of the conditional pdf will be different for different types of channels [23]. Therefore, a profound understanding of $f_{Y|X}$ is indispensable when designing error correction codes, optimizing modulation schemes, or improving the overall reliability of communication systems.

As is well-known, the pdf for a pure AWGN channel can be expressed as

(3) $$\begin{array}{cc} f_{Y|X}\left(y|x\right) & =\frac{1}{\pi N_{0}}\exp\left(-\frac{{\left|\left|y-x\right|\right|}^{2}}{N_{0}}\right), \end{array}$$

where $\left|\left|\cdot \right|\right|$ represents the Euclidean norm. Due to discrete-probability transmitted signals, the pdf $f_{Y}$ can thus be expressed as

(4) $$\begin{array}{cc} f_{Y}\left(y\right) & =\sum_{i=1}^{M}f_{Y|X}\left(y|x_{i}\right)f_{X}\left(x_{i}\right). \end{array}$$

For GCS, we focus on the case of discrete and equi-probable transmitted signals, i.e., $f_{X}\left(x_{i}\right)=\frac{1}{M}$ for any *i*. Conversely, for PCS transmission, the symbols are not distributed with equal likelihood, and $f_{X}\left(x_{i}\right)$ is to be optimized.

In brief, the MI calculation using (2) depends on the specific channel models and is closely related to the constellation distributions. However, in most practical cases, the underlying joint or conditional pdf of the channel is usually unknown or not estimated. Therefore, MINE is considered as a possible solution to address the issue of accurate MI estimation in the following.

### 2.3. The MINE Principle

For the MINE principle, the Kullback–Leibler (KL) divergence $D_{\mathrm{KL}}$, also known as information divergence or relative entropy, is also considered here as an asymmetric measure of the difference between two probability distributions [24]. In general, the MI between *X* and *Y* is equivalent to the KL divergence between the joint distribution $f_{\mathrm{XY}}$ and the product of the marginals $f_{X}⊗f_{Y}$. Therefore, the calculation of MI can be achieved by deriving the KL divergence, that is

(5) $$\begin{array}{cc} I\left(X;Y\right) & =\int_{X\times Y}\log\frac{f_{XY}}{df_{X}⊗f_{Y}}df_{XY}\\ \; & =E_{f_{XY}}\left[\log\frac{df_{XY}\;}{df_{X}⊗f_{Y}}\right]\\ \; & =D_{\mathrm{KL}}\left(f_{XY}\;∥\;f_{X}⊗f_{Y}\right). \end{array}$$

Note that the MINE primarily centers on the Donsker–Varadhan (DV) representation, which is the special case of the dual representation of KL divergence when the function is xlogx [25]. The Donsker–Varadhan representation is specifically expressed as ([18] Equation (5))

(6) $$D_{\mathrm{KL}}\left(f_{XY}∥f_{X}⊗f_{Y}\right)=\underset{T:\Omega \to R}{\sup}E_{f_{XY}}\left[T\right]-\log\left(E_{f_{X}⊗f_{Y}}\left[e^{T}\right]\right),$$

where *T* is a function in the set of all functions R, $\left.T:\Omega \to R\right.$ satisfying the integrability constraints of the theorem, sup means the upper bound of all measurable functions *T*, and *e* is the natural logarithmic base. In comparison with the *f*-divergence representation, the Donsker–Varadhan representation is demonstrated to offer stronger bounds, thereby providing a more rigorous estimation for high-dimensional variables [18,26].

The idea here is to assume a function family $\left.T_{\theta }:X\times Y\to R\right.$ parametrized by a DNN with parameters θ∈Θ, and employing a gradient descent strategy to optimize the DNN and obtain the optimal $T_{\theta optim}$. The relationship between the neural MI measure and the real MI is thus defined as

(7) $$I_{\Theta }\left(X,Y\right)\leq I\left(X;Y\right),$$

where the neural information estimator with parameter θ∈Θ is defined as

(8) $$I_{\Theta }\left(X,Y\right)=\underset{\theta \in \Theta }{\sup}E_{f_{XY}}\left[T_{\theta }\right]-\log\left(E_{f_{X}⊗f_{Y}}\left[e^{T_{\theta }}\right]\right).$$

This converts the exact estimation problem into a parameter optimization problem, employing the DNN to iteratively approximate and maximize the MI between the input and output samples of the channel. In particular, the expectation therein is approximated by empirical samples from joint or marginal distributions. MINE can effectively estimate the MI and capture the nonlinear dependencies without clear knowledge of the channel density function.

## 3. Constellation Shaping Design

**Figure 1:**
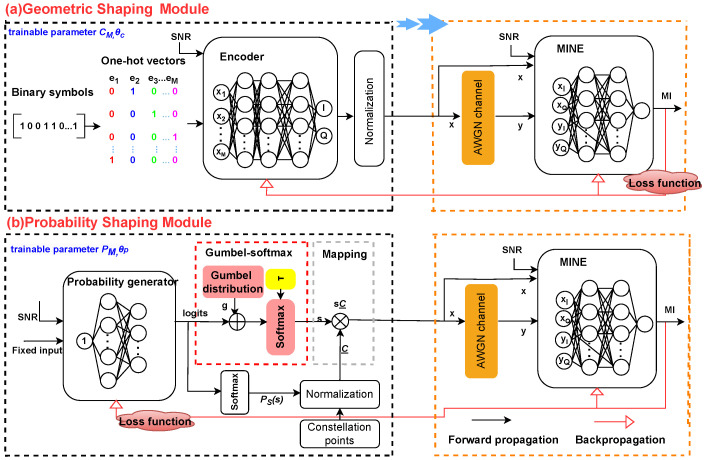
Block diagrams of the proposed MINE-based constellation shaping system.

In this section, we present a MINE-driven constellation shaping system that incorporates MINE into GCS, PCS, and JCS designs. As shown in Figure 1, the system consists of a geometric shaping module and a probability shaping module, which are employed to optimize the geometric or probabilistic distribution of constellation points. On this basis, we further design a joint shaping scheme called MINE-JCS that simultaneously optimizes the constellation point locations and probabilities. The MINE-JCS scheme uses the optimized geometric locations of the GCS to input into the PCS training for joint optimization, to achieve better noise tolerance and MI performance.

Here, $C_{M}$ and $\theta_{C}$ denote the trainable parameters of the encoder and MINE estimator for GCS, while the trainable parameters denoted as $P_{M}$ and $\theta_{P}$ represent the probability mapping and MI estimation in PCS. The overall goal of the training phase is to maximize Equation (8) for the AWGN channel over the weight set $w_{1}$ = {$C_{M}$, $\theta_{C}$} or $w_{2}$ = {$P_{M}$, $\theta_{P}$}. After training convergence, the weights for the encoder and probability generator are fixed, and the Monte Carlo method is used for validation. For JCS optimization, $J_{M}$ and $\theta_{J}$ are the trainable parameters for joint mapping of the location and probability and MI estimation, i.e., the weight set $w_{3}$ = {$J_{M}$, $\theta_{J}$}.

### 3.1. The MINE-Based MI Estimation

Based on the MINE principle, we employ a backpropagation algorithm in the optimization. It is very important to calculate the gradient of the loss function and update the model parameters accordingly. When training the model, we set the negative value of MI measurement as the loss function, that is

(9) $$\begin{array}{cc} Loss & =-I_{\Theta }\left(X,Y\right)\\ \; & =-\left\{\underset{\theta \in \Theta }{\sup}E_{f_{XY}}\left[T_{\theta }\right]-\log\left(E_{f_{X}⊗f_{Y}}\left[e^{T_{\theta }}\right]\right)\right\}. \end{array}$$

In simulations, the natural logarithm (base *e*) is typically used instead of the base 2 logarithm, which does not affect the optimization result. With this specific loss function, MINE explicitly optimizes the front-end encoder with the expert information of the MI and is considered to outperform an E2E learning setup. It should be noted that MINE is only involved in the training process.

### 3.2. MINE-GCS Design Architecture

As shown in Figure 1a, the proposed MINE-based GCS architecture is composed of two DNN-based modules, the encoder and the MINE. The goal is to maximize the MI between the channel input *x* and output *y* by optimizing the locations of the constellation symbols.

In order to learn the geometric constellation shape, a random string of raw bits is first generated and subsequently mapped to constellation symbols, following both gray and natural encoding rules. To be clear, the raw bits have a total size of $B_{s}$ × *M*, where $B_{s}$ denotes the training batch size. Then, we convert the *M*-ary constellation symbols into corresponding one-hot code vectors. The element of the one-hot vector is defined as

(10) $$\begin{array}{c} e_{v_{i}}\left[j\right]=\left\{\begin{array}{cc} 1, & \mathrm{if}\;\;i=j\\ 0, & \mathrm{otherwise} \end{array}\right.\;\;\;\;\;\;\;j=0,\ldots ,M-1, \end{array}$$

where $v_{i\;}$ denotes the *i*-th symbol of the constellations, and $e_{v_{i}}\left[j\right]$ is the *j*-th vector element corresponding to the symbol $v_{i}$. Next, the generated one-hot vectors are fed into the encoder and modulated into the *I*/*Q* components. Note that the normalization before the channel places an average power constraint on the learned constellation, which is to ensure the expected energy is unity. The normalized modulated symbol is denoted by $x_{IN}$ and $x_{QN}$.

**Figure 2:**
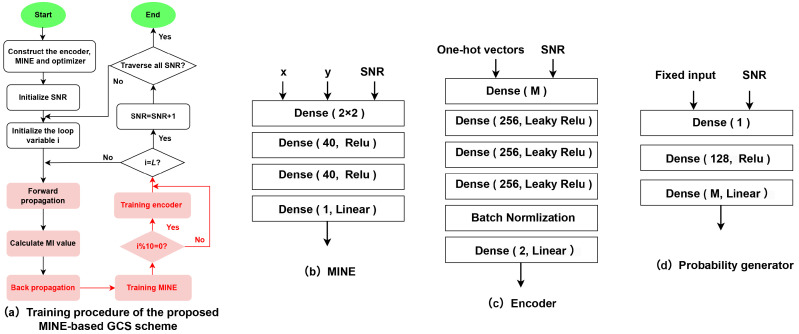
Training procedure of the proposed MINE-GCS scheme and the specific structure of DNNs.

As shown in Figure 1, the modulated symbol is propagated through the set channel, and the received symbol $y_{k}$ can be obtained based on Equation (1). $x_{k}$ and $y_{k}$ at both ends of the channel are input into the MINE to output the estimated MI values. For the proposed MINE-based system, instead of approximating the channel probability distribution itself, the optimization of the parameter $\theta_{C}$ is performed by iteratively approximating and maximizing the MI between the samples of the channel input and output. At the same time, the encoder network is trained by backpropagation, and the constellation positions $C_{M}$ can be updated for every SNR. In this way, the two parameters are trained alternately until convergence, and the maximum MI can be finally obtained. This benefits from the use of DL in channel encoding, without requiring explicit knowledge of the channel transfer probability. The training procedure of the proposed MINE-GCS scheme is shown in Figure 2a.

The encoder, MINE, and optimizer are first constructed. A total of *L* iteration cycles are trained. In each training cycle, the constellation locations to be optimized for each SNR and the MI value are calculated based on the trained MINE network.

### 3.3. MINE-PCS Design Architecture

As depicted by Figure 1b, the proposed MINE-based PCS training structure is composed of two DNN-based modules, i.e., the probability generator and MINE. The probability generator network first outputs the weight vector of constellation points, which is the logits to be optimized in Figure 1b. Then, a softmax activation function is applied to the logits to generate the discrete probability distribution $P_{S}\left(s\right)$, which is a key variable trained indirectly. Each element represents the probability of the corresponding category. The backpropagation algorithm is deployed in the differentiable layer to allow efficient calculation of the parameter gradient. Note that the challenge of performing probabilistic shaping using DL-based algorithms lies in designing a trainable, differentiable sampler. That is to say, in order to implement the DNN-based sampling mechanism, the problems we need to tackle are twofold: first, how to devise a method for sampling from a discrete distribution, and second, how to ensure the entire process is differentiable.

**Figure 3:**
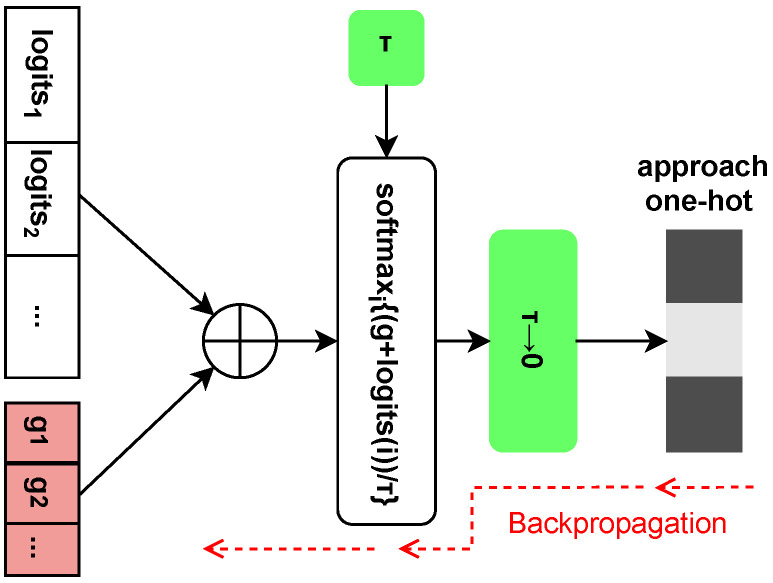
Specific Gumbel-softmax structure.

As we know, the Gumbel-max trick provides a simple and efficient way to draw symbols from the categorical distribution, allowing us to obtain an infinite number of one-hot vectors which obey the original discrete distribution $P_{S}\left(s\right)$. We thus can achieve the purpose of sampling from the discrete distribution. However, the Gumbel-max method cannot be used to update the network because the arg max operator in the Gumbel-max trick is not differentiable. Therefore, the Gumbel-softmax trick in [27] is leveraged to address these issues in our work, by replacing the non-differentiable samples from the classification distribution with novel differentiable samples from the Gumbel-softmax distribution. Note that the Gumbel-softmax is implemented in Figure 3, and the softmax function is employed as a differentiable, continuous approximation to the arg max. Thus, we have the output symbols s, derived as

(11) $$\begin{array}{cc} s & =\;\mathrm{softmax}\left(\frac{g_{i}+logits}{\tau }\right)\\ \; & =\;\frac{\exp\left(\left(g_{i}+logits\right)/\tau \right)}{\sum_{j}\exp\left(\left(g_{j}+logits\right)/\tau \right)}, \end{array}$$

where the Gumbel noise variables denoted as $g_{i}=-\log\left(-\log\left(u_{i}\right)\right)$ are independent and identically distributed samples drawn from a standard Gumbel distribution Gumbel(0,1), which $u_{i}$ obeys Uniform(0,1). This will be introduced to return unfixed results for samples. In addition, τ indicates the non-negative scalar temperature and is used as the annealing parameter, which controls the smoothness of the softmax function. Set τ→0 in the training set, enabling the samples from the Gumbel-softmax method to approach the one-hot vectors. As a result, the Gumbel-softmax distribution thus becomes closer to $P_{S}\left(s\right)$. Up to this point, gradient update of discrete distributions with backpropagation is allowed.

According to Figure 1b, we treat the probability generator network and Gumbel-softmax module together as a whole sampler, with $P_{M}$ as the optimized parameter of the sampler. For the mapping part, $C=\{c_{1},c_{2},\ldots ,c_{M}\}$ represents the set of standard constellation points with corresponding probabilities $P_{M}=\left\{p_{1},p_{2},\ldots ,p_{M}\right\}$. The normalized symbol $\underline{C}=\left\{{\underline{c}}_{1},{\underline{c}}_{2},\ldots ,{\underline{c}}_{M}\right\}$ can be expressed as

(12) $${\underline{c}}_{n}=c_{n}/\sqrt{\sum_{i=1}^{M}p_{i}c_{i}^{2}}.$$

By taking the product of $\underline{C}$ and a one-hot-approximated vector s of length *M*, one can select $s\underline{C}$ as the set of constellation points for the MINE-PCS scheme.

### 3.4. MINE-JCS Design Architecture

Note that MINE-JCS combines MINE-GCS and MINE-PCS to achieve better performance by simultaneously optimizing the locations and probabilities of constellation points.

Given that the JCS stems from the GCS and PCS, this paper does not elaborate on its framework structure here. It is particularly emphasized that the only power constraint is based on the optimized geometric positions $C_{g}=\left\{c_{g1},c_{g2},\ldots ,c_{gM}\right\}$. The normalized symbol ${\underline{C}}_{g}=\left\{{\underline{c}}_{g1},{\underline{c}}_{g2},\ldots ,{\underline{c}}_{gM}\right\}$ can be expressed as

(13) $${\underline{c}}_{gn}=c_{gn}/\sqrt{\sum_{i=1}^{M}p_{i}c_{gi}^{2}}.$$

MINE-JCS also uses the Gumbel-softmax trick to avoid non-differentiable samples.

## 4. Numerical Results

This section shows the simulated MI performance of the designed constellations with AWGN, under various SNRs and modulation orders. Detailed comparisons with the standard QAM and novel DL-based designs are given to verify the advantages of our schemes.

### 4.1. Experimental Settings

#### 4.1.1. DNN Architecture and Hyper-Parameters

The GPU experiments in this work mainly have been implemented on i7-12700H, and Nvidia GeForce 3050Ti graphics card with Pytorch powered with CUDA 11.3 [28]. Taking the 16-ary design as an example, Table 1 and Table 2 give the training hyperparameters used for the MINE-GCS and MINE-PCS learning, respectively. Note that the MINE-JCS scheme was based on the combination of both the training process and learning parameters.

The DNN-based encoder comprised an input layer with *M* neurons, an output layer with 2 neurons, and 3 hidden layers each containing 256 neurons, as proposed in Figure 2c. The Leaky ReLU was used as the activation function for the encoder. As seen in Figure 2b, the MINE structure was further improved based on [30], which had two inputs, with each input featuring two linear layers and a ReLU activation function. The overall MINE architecture comprised two hidden layers, each housing 40 neurons. The input dimension was 2×2, and the output layer was a linear layer with one output dimension. Note that the output neurons of the encoder depict the in-phase and quadrature (*I*/*Q*) components of the modulated signal, and the input neurons of MINE depict the normalized two-dimensional *I*/*Q* components. In Figure 2d, the DNN-based probability generator was composed of an input layer with 1 neuron, an output layer with *M* neurons, and one hidden layer with 128 hidden neurons. The ReLU was used as the activation function for the probability generator. The MINE was identical to the one described in the aforementioned geometric scheme, with its trainable parameter denoted as $\theta_{P}$.

#### 4.1.2. Comparative Methods

**Figure 4:**
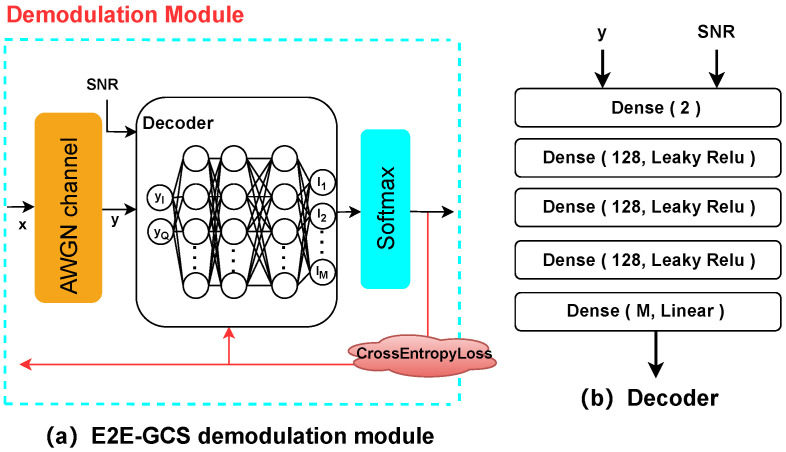
Structure of the E2E-GCS system for demodulation.

In addition to comparing with the conventional QAMs as baselines, some well-known DL-based schemes were considered here, including the E2E-based GCS (abbreviated as “E2E-GCS”), and the E2E-based PCS and JCS schemes proposed previously in [13] (abbreviated as “2019Stark-PCS” and “2019Stark-JCS”, respectively). All the considered schemes were trained only with pure AWGN but evaluated in all channel conditions, validating the reliability of our MINE-driven designs in various environments. To be specific, we had the following:

- E2E-GCS: We first considered a comparative E2E-GCS structure, which included the modulation and demodulation modules to be trained. The modulation module was consistent with the modulation module in Figure 1a. For the demodulation module, the decoder DNN had an input layer with 2 neurons, an output layer with *M* units, and 3 hidden layers, each containing 128 units. Moreover, the Leaky ReLU activation function and Adam optimizer were used in the training. The specific settings of the E2E-GCS demodulation module and decoder network are shown in Figure 4. The symbol-metric demapper was implemented as a DNN-based categorical classifier with trainable parameter $\theta_{D}$. Categorical cross-entropy (CE) was used as the loss function to update the model parameters, which is given by ([20] Equation (3)).
  (14) $$\begin{array}{cc} L_{CE} & =-\sum_{i}x_{i}\log\left(S_{i}\right)\\ \; & =-\sum_{i}x_{i}\log\left\{\frac{\exp\left(l_{i}\right)}{\sum_{j=1}^{M}\exp\left(l_{j}\right)}\right\}, \end{array}$$
  where $x_{i}$ represents the input vector of the encoder, $S_{i}$ represents the softmax function, and $l_{i}$ is the score generated by the decoder.

- 2019Stark-PCS and 2019Stark-JCS: Recently, ref. [13] also proposed a trainable E2E communication system for a AWGN channel, which corresponds to the comparative E2E-based 2019Stark-PCS and 2019Stark-JCS schemes we employ here. In order to train the modulator and demodulator together, the complex categorical CE loss function was set to be equivalent to maximizing the MI of the channel inputs *X* and outputs *Y*, while minimizing the KL divergence between the true posterior distribution f(x|y) and the distribution learned by the receiver. Note that the autoencoder in [13] was mainly leveraged to perform probabilistic shaping, and the influence of source entropy was removed in the PCS training to avoid the unwanted effect of source entropy reduction caused by minimizing the categorical CE.

In comparison, our MINE-driven scheme employed DV-based optimization to estimate and maximize the MI, and avoided training of the demodulator, which is simpler and more straightforward in a practical implementation.

#### 4.1.3. Complexity Analysis

Table 3 gives a complexity comparison of our proposed schemes with the E2E-GCS and 2019Stark-PCS schemes, including their specific floating point operations (FLOPs), the number of parameters, and the required training time per SNR. Note that we only consider 16QAM for a simple illustration here, and the time consumption was tested on the GPU version of PyTorch. It is shown that our MINE-PCS had the least numbers of FLOPS and parameters, while our MINE-GCS had the minimum training time per SNR. And the training time for both MINE-driven schemes was much lower than the other two schemes. We can explain this phenomenon from the perspective of a complexity analysis of the DNN structures involved in each scheme. As such, Table 4 gives the specific numbers of FLOPs and parameters of the six NN models involved in the four shaping schemes. We can see that the MINE, the probability generator, and the encoder (2019Stark-PCS) had a much smaller number of FLOPS and parameters than the other modules. More importantly, since our proposed schemes do not involve a decoder, the training time required was much less than that of the E2E and 2019Stark-PCS schemes. This is the reason why our MINE-GCS and MINE-PCS required less time and computational complexity than the E2E-GCS and 2019Stark-PCS, respectively.

### 4.2. MINE-Driven Optimized Constellations

**Figure 5:**
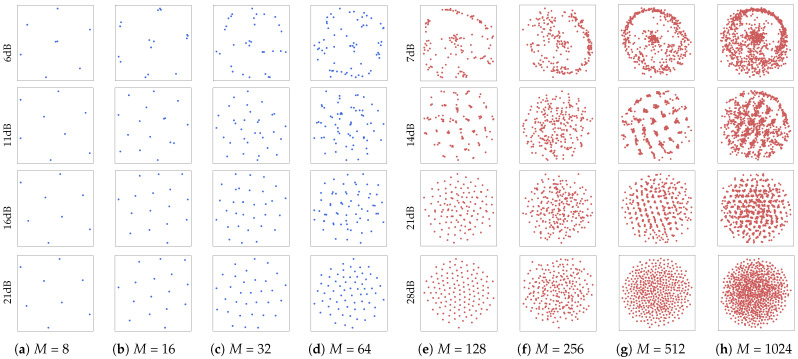
Optimized constellations with respect to *M*-ary for MINE-GCS at several SNRs, for: (**a**) 8-ary, (**b**) 16-ary, (**c**) 32-ary and (**d**) 64-ary, (**e**) 128-ary, (**f**) 256-ary, (**g**) 512-ary and (**h**) 1024-ary constellations, respectively.

This subsection presents the MINE-driven optimized constellations of different orders under the different channel conditions. Note that these were obtained by MI maximization with respect to the varying SNR in the AWGN training channel. Figure 5 shows the MINE-GCS constellations concerning *M* from 8 to 1024 at different SNRs. In these figures, the horizontal and vertical coordinates represent the normalized energy of the signal in the *I*/*Q* components. Note that lower-order constellations, e.g., 8-ary in Figure 5a, reached a stable state first, and then changed little with SNR increases. To better visualize the changes in point locations, a higher SNR is required for constellations of higher orders. For a given order, e.g., 128-ary in Figure 5e with varying SNRs, it is obvious that the constellation points are clustered in groups with low SNRs. With the increase in the SNR, the minimum Euclidean distance (MED) between any adjacent points increased gradually, and the constellation points exhibited stronger circular symmetry. This observation suggests that these points possessed greater tolerance to AWGN at higher SNRs.

**Figure 6:**
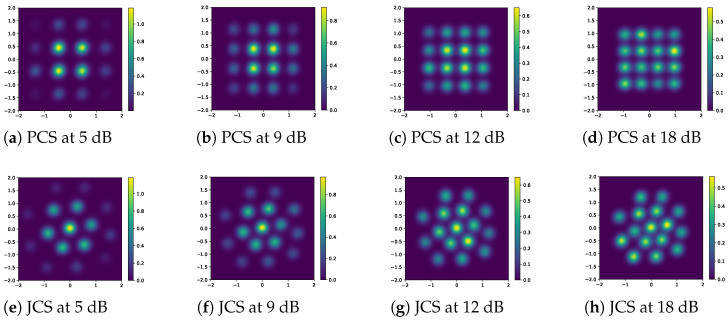
The 16-ary optimized constellation points for MINE-PCS and MINE-JCS at several SNRs.

Figure 6 displays the 16-ary constellation diagrams of the MINE-PCS and MINE-JCS at different SNRs. For the MINE-PCS optimization, the shape remained the same as the traditional QAM. The MINE-JCS optimized the probability shaping together with the location shaping. The difference was that the larger the size of the constellation point, or the higher its brightness weight, the greater the probability of transmission for that specific point. We can observe that for low SNRs, e.g., below 9 dB for the 16-ary design, the probability of the central constellation points was higher, while the probability of the surrounding points was lower. The learned distribution shape was thus similar to a three-dimensional inverted bell curve. This is in accordance with the fact that simulating a Gaussian-like constellation shape can enhance the MI in pure AWGN [13]. As the SNR increased, the weight of the surrounding constellation points gradually increased, and approached that of the central points. As Figure 6d,h show, their distributions tended to become more uniform for high SNRs.

**Figure 7:**
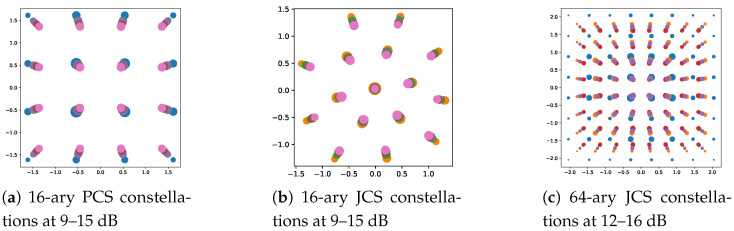
Constellation trajectories for *M*-QAM at varying SNRs.

Moreover, Figure 7 depicts the constellation trajectories of *M*-ary MINE-PCS/JCS with varying SNRs, which helps to show how the proposed scheme learned the shaping during optimization. We consider *M* = 16 and 64 as examples and select the SNR segments that are of practical interest. Figure 7a,b illustrate the trajectories of the 16-ary PCS and JCS constellation maps at 9–15 dB, respectively. Figure 7c shows the trajectories of the 64-ary constellation maps at 12–16 dB. For the MINE-PCS optimization in Figure 7a, it is interesting to see that, in addition to varying sizes, the normalized constellation trajectory moves towards the center as the SNR increases, due to power normalization. That is, to maintain a constant average energy, the surrounding points with the gradually increased probability had to move closer to the center of the constellation diagram for energy balance. Similar and more pronounced trends can be observed in Figure 7b,c, where the MINE-JCS optimization helped improve the transmission performance in the entire SNR region.

**Figure 8:**
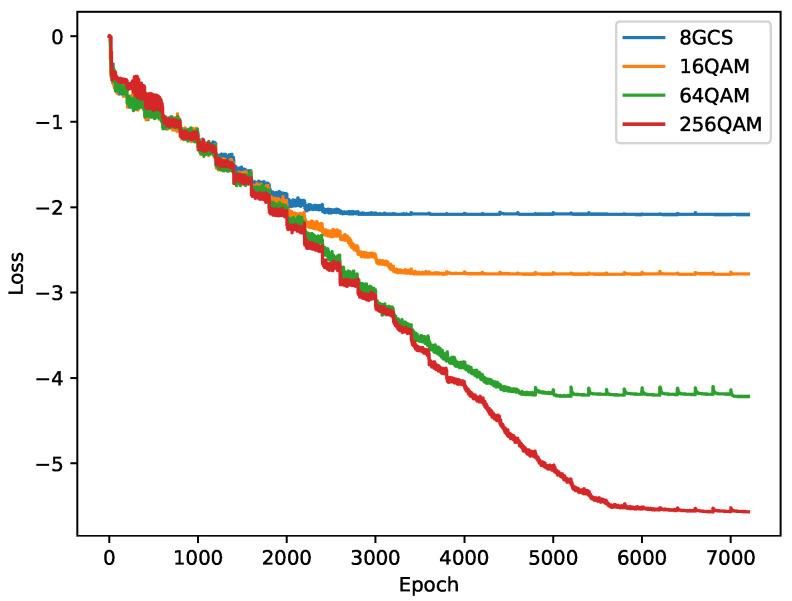
Convergence analysis for the MINE-based GCS training.

To further verify the reliability of the experiments, we present the convergence curves of the MINE structure training in Figure 8, where the loss value has a $-\frac{\log e}{\log2}$ scaling relationship with the actual MI. Since the convergence patterns of the MINE-based schemes were similar, we only show the convergence of MINE-GCS at different modulation orders. Note that the SNR was trained from 0 to 35 dB with 200 epochs per SNR for simple illustration. The loss curve shows a step-down trend and stabilized after reaching a certain SNR for a given order. As expected, the training of the lower-order GCS converged faster and could achieve its minimum loss at a lower SNR. This implies that the lower-order constellations could achieve the maximum MI at lower SNR, which is consistent with the phenomenon in Figure 5.

### 4.3. MI Performance Analysis for AWGN Test Channel

**Figure 9:**
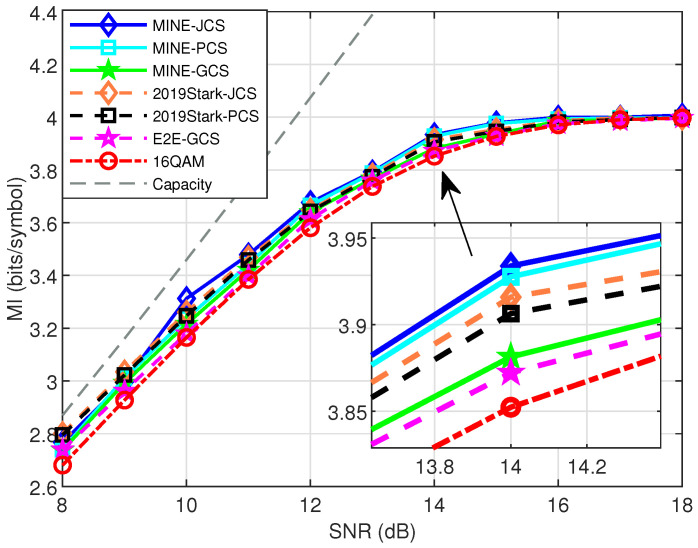
MI performance of MINE-based schemes and other popular schemes for 16-ary design over AWGN test channel.

We compared the optimized constellations with several baseline schemes, to evaluate the MI performance for the AWGN test channel. As shown in Figure 9 for 16-ary constellations, the overall MI performance can be sorted as “MINE-JCS > MINE-PCS > 2019Stark-JCS > 2019Stark-PCS > MINE-GCS > E2E-GCS”. And they were all much better than that of the unshaped 16QAM within the studied SNR range. It was expected that the MINE-JCS would perform the best, since the positions and probabilities of the constellation points were jointly optimized. It is interesting to see that even the MINE-PCS performed better than the 2019Stark-JCS and 2019Stark-PCS. This may be because our MINE-driven scheme did not need to train the decoder, thus avoiding the loss caused by decoder training. To be specific, at SNR = 14 dB, the 16-ary MINE-PCS scheme exhibited MI gains of approximately 0.02 and 0.04 bits/symbol compared to the 2019Stark-JCS and 2019Stark-PCS, respectively. For 64-ary and higher-order constellations, our MINE-based methods undoubtedly had a superior MI performance than the unshaped QAMs at all tested SNRs. With the increase in SNR, the MI of our MINE-based schemes gradually approached the system capacity, and performed similarly to that of the E2E-based 2019Stark-JCS/PCS schemes, which are omitted here.

**Figure 10:**
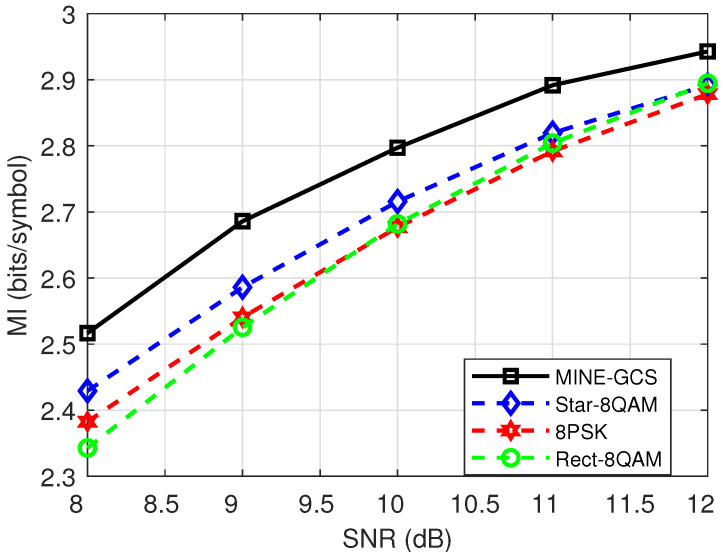
MI performance comparison between 8-ary MINE-GCS and different shaped 8QAM schemes over AWGN test channel.

For the MINE schemes considered, the MINE-PCS performed much better than the MINE-GCS for pure AWGN. For example, in Figure 9, the MINE-PCS has an approximately 0.05 bits/symbol MI gain over MINE-GCS at 14 dB. According to the order of “MINE-JCS > MINE-PCS > MINE-GCS ”, we further selected the MINE-GCS scheme with relatively poor performance to compare with a variety of unshaped 8QAMs under pure AWGN, as Figure 10 shows. The simulations considered the SNR range from 8 to 12 dB of practical interest, for better visualization. It can be observed that the MINE-GCS scheme performed remarkably better than the three unshaped 8QAMs. For example, MINE-GCS had about 0.1 bits/symbol MI gain over Star-8QAM at SNR = 8 dB. This also verified the MI performance advantage of our proposed schemes for low-order modulations with AWGN.

**Figure 11:**
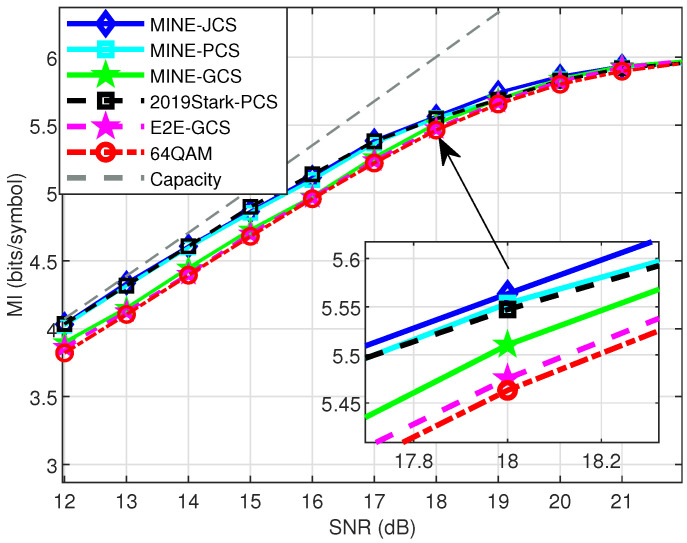
MI performance of MINE-based schemes and other popular schemes for 64-ary design over AWGN test channel.

As shown in Figure 11, for the 64-ary constellation points, the MINE-JCS, MINE-PCS, and 2019Stark-PCS schemes had significantly better MI performance than the other three schemes at relatively low SNRs, such as before SNR = 18 dB. With the increase in SNR, the performance of the various shaping schemes was similar, and the system capacity was gradually approached. In particular, the NNs had a certain degree of randomness, and the MI performance we tested was not stable. When SNR = 16 dB, the 2019Stark-PCS performed best, with an MI gain of approximately 0.03 and 0.04 bits/symbol compared to the MINEJCS and MINE-PCS, respectively. And the 2019Stark-PCS method was significantly better than the MINE-GCS solution. For SNR = 18 dB, MINE-JCS performed better, with about a 0.1 bits/symbol MI gain compared to unshaped 64QAM. In addition, whether for 16-ary or 64-ary, the conventional E2E-GCS and the unshaped QAM schemes consistently fell short in competitiveness.

### 4.4. MI Performance Analysis in Test Channel

**Figure 12:**
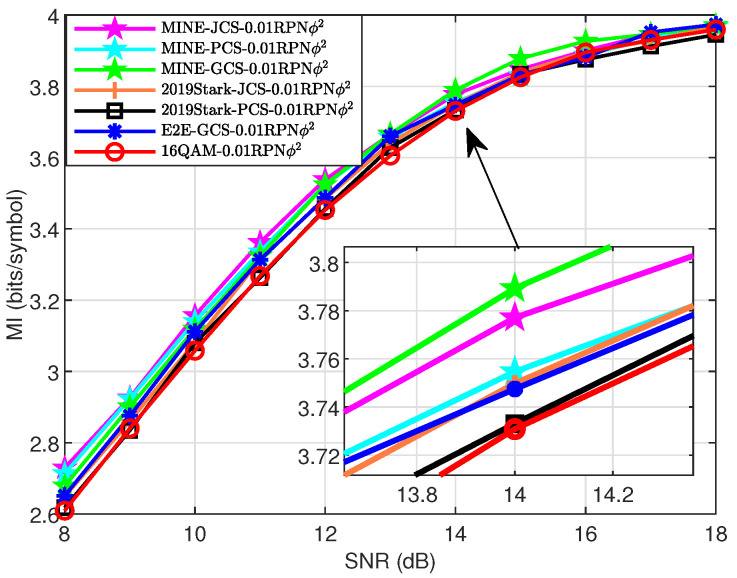
MI performance of different schemes for 16-ary constellations over RPN test channel with $\sigma^{2}=0.01$ rad^2^.

Although this study primarily focused on AWGN channels, we expect to provide a general MI optimization framework that can adapt to complex channels. Therefore, we conducted a preliminary evaluation of the MINE scheme by applying the shaping network, trained under AWGN conditions, to complex channels with varying levels of phase noise and fading, assessing its adaptability. In Figure 12, we verify the robustness of the proposed MINE scheme under the condition of $\sigma^{2}=0.01$ rad^2^ on the AWGN+RPN test channel. It can be observed that the MI performance of the MINE schemes consistently surpassed that of the 16QAM, E2E-GCS, and 2019Stark-PCS/JCS schemes throughout the whole SNR region. Additionally, MINE-JCS performed the best at low SNR, followed by the MINE-PCS scheme. Meanwhile, as the SNR increased, the MINE-GCS became less sensitive to the phase noise beginning at approximately 13 dB, which led to higher MI. From the magnification at SNR = 14 dB, we can see that the MINE-GCS achieved MI gains of approximately 0.02 and 0.04 bits/symbol, respectively, compared to the MINE-JCS and 2019Stark-JCS schemes.

**Figure 13:**
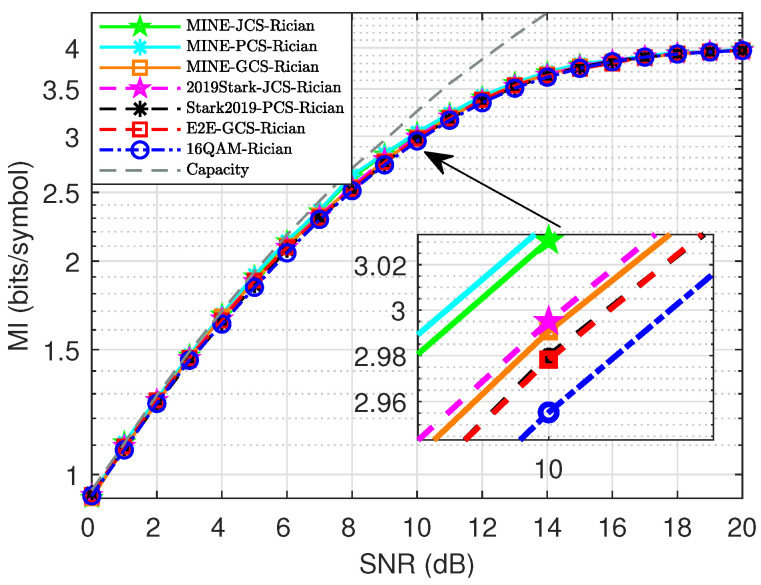
MI performance comparison for 16-ary constellations with Rician test channel.

As shown in Figure 13, the MI performance of several schemes was compared and analyzed using Rician fading with a Rician factor K = 5. It is shown that our MINE-based methods always had a better MI performance, especially in the low to medium SNR range. Specifically, in Figure 13 for 16-ary constellations, the MI performance of MINE-PCS and MINE-JCS are very close, and they significantly outperformed the other comparable schemes. For example, at SNR = 10 dB for the 16-ary design, the MINE-PCS exhibited MI gains of approximately 0.0437 and 0.0588 bits/symbol, compared to the 2019Stark-JCS and 2019Stark-PCS, respectively. For the 64-ary design at 12 dB, the MINE-PCS had an MI gain of approximately 0.1419 bits/symbol over 64QAM. It is worth noting that our MINE-PCS performed much better than MINE-GCS in multipath fading. This implies that the PCS scheme which optimized the distribution probability is more beneficial to the design with fading, and thus dominated for the joint scheme.

## 5. Conclusions

A MINE-driven constellation shaping optimization scheme was proposed, which showed superior MI performance under different channel conditions. The key fact is that the MINE provides a general DNN-based MI calculation, which avoids the requirement of explicit channel information that traditional methods struggle with. The proposed MINE-based schemes were much more robust compared to several baseline schemes, including QAMs, E2E-GCS, and 2019Stark-JCS, etc. It is worth noting that with the increase in constellation order, the capacity gain increased slightly, MINE-JCS particularly performed better. Note that the system only optimizes the transmitter end and does not need to train the decoder, thereby avoiding the loss and complexity involved in decoding. The MINE-driven solution avoids a fixed rate distribution matcher and provides flexible constellation adaptation through neural networks. It has stronger versatility and scalability than constellation shaping models such as enumeration classes or genetic algorithms based on non-DL learning. In the future, we intend to extend the application of this DL-based approach to complex channel environments, which will enable us to conduct more comprehensive robustness evaluations and provide stronger empirical evidence of the system’s performance under various practical conditions.

## Figures and Tables

**Table 1:** Training parameters for the MINE-GCS scheme.

Hyperparameters	Values
Number of hidden layers	Encoder (3), MINE (2)
Number of hidden neurons	Encoder (256), MINE (40)
Loss function	Equation (9)
Learning rate	0.01
Batch size	800
Epoch	200
Optimizer	Adam [29]
Training cycle	Encoder (every 10 epoch),
	MINE (every 1 epoch)

**Table 2:** Training parameters for the MINE-PCS scheme.

Hyperparameters	Values
Number of hidden layers	Probability generator (1),
	MINE (2)
Number of hidden neurons	Probability generator (128),
	MINE (40)
Loss function	Equation (9)
Learning rate	0.01
Batch size	5000
Epoch	2000
Optimizer	Adam
Training cycle	Probability generator (every 1
	epoch), MINE (every 1 epoch)

**Table 3:** Complexity comparison of different shaping methods.

	FLOPs	Params	Training Time for per SNR
MINE-GCS	0.1391 M	0.14 M	0.8380 s
E2E-GCS	0.1708 M	0.172 M	70.6799 s
MINE-PCS	5576	5881	1.4815 s
2019Stark-PCS	0.0177 M	0.0181 M	6.1650 s

**Table 4:** Complexity analysis of neural network structures.

	FLOPs	Params
MINE	3400	3561
Probability Generator	2176	2320
Encoder (MINE- and E2E-GCS)	0.1357 M	0.1365 M
Decoder (E2E-GCS)	0.0171 M	0.0172 M
Encoder (2019Stark-PCS)	640	772
Decoder (2019Stark-PCS)	0.017 M	0.0173 M

## Data Availability

The raw data supporting the conclusions of this article will be made available by the authors on request.
